# Early infiltration of p40IL12^+^CCR7^+^CD11b^+^ cells is critical for fibrosis development

**DOI:** 10.1002/iid3.114

**Published:** 2016-07-21

**Authors:** Tarcio Teodoro Braga, Matheus Correa‐Costa, Hatylas Azevedo, Reinaldo Correia Silva, Mario Costa Cruz, Maira Estanislau Soares Almeida, Meire Ioshie Hiyane, Carlos Alberto Moreira‐Filho, Marinilce Fagundes Santos, Katia Regina Perez, Iolanda Midea Cuccovia, Niels Olsen Saraiva Camara

**Affiliations:** ^1^ Laboratory of Transplantation Immunobiology, Department of ImmunologyInstitute of Biomedical Sciences IV, University of São Paulo (USP)São PauloBrazil; ^2^ Department of PediatricsFaculdade de Medicina da Universidade de São Paulo (FMUSP)São PauloBrazil; ^3^ Department of Cellular Biology–Institute of Biomedical Sciences University of São Paulo (USP)São PauloBrazil; ^4^ Department of Biochemistry–Institute of ChemistryUniversity of São Paulo (USP)São PauloBrazil; ^5^ Laboratory of Clinical and Experimental Immunology, Division of NephrologyFederal University of São Paulo (UNIFESP)São PauloBrazil; ^6^ Renal Pathophysiology Laboratory (LIM16)Faculty of Medicine, University of São PauloSão PauloBrazil

**Keywords:** Collagen, pro‐inflammatory macrophages, renal fibrosis

## Abstract

**Introduction:**

Macrophages are heterogeneous and thus can be correlated with distinct tissue outcomes after injury. Conflicting data have indicated that the M2‐related phenotype directly triggers fibrosis. Conversely, we hypothesize here that the inflammatory milieu provided by early infiltration of pro‐inflammatory macrophages dictates tissue scarring after injury.

**Methods and Results:**

We first determined that tissue‐localized macrophages exhibit a pro‐inflammatory phenotype (p40IL12^+^CCR7^+^CD11b^+^) during the early phase of a chronic injury model, in contrast to a pro‐resolving phenotype (Arg1^+^IL10^+^CD206^+^CD11b^+^) at a later stage. Then, we evaluated the effects of injecting macrophages differentiated in vitro in the presence of IFNγ + LPS or IL4 + IL13 or non‐differentiated macrophages (hereafter, M0) on promoting inflammation and progression of chronic injury in macrophage‐depleted mice. In addition to enhancing the expression of pro‐inflammatory cytokines, the injection of M (IFNγ + LPS), but not M (IL4 + IL13) or M0, accentuated fibrosis while augmenting levels of anti‐inflammatory molecules, increasing collagen deposition and impairing organ function. We observed a similar profile after injection of sorted CCR7^+^CD11b^+^ cells and a more pronounced effect of M (IFNγ + LPS) cells originated from Stat6^−/−^ mice. The injection of M (IFNγ + LPS) cells was associated with the up‐regulation of inflammation‐ and fibrosis‐related proteins (Thbs1, Mmp7, Mmp8, and Mmp13).

**Conclusions:**

Our results suggest that pro‐inflammatory macrophages promote microenvironmental changes that may lead to fibrogenesis by inducing an inflammatory milieu that alters a network of extracellular‐related genes, culminating in tissue fibrosis.

## Introduction

The development of fibrosis is closely associated with the occurrence of a previous acute injury episode. Although initially beneficial, this process becomes pathological if it remains continuous, resulting in substantial remodeling of the extracellular matrix (ECM) and formation of a permanent scar 1. Depending on the inflammatory response elicited immediately after acute injury, infiltrating leukocytes, and their products result in the resolution or progression of lesions 2, 3. Among infiltrating cells, macrophages are relevant in tissue regeneration and the development of fibrosis 4, 5, 6. However, characterizing macrophage subtypes in the context of the fibrotic process rather than only describing the presence of macrophages in tissue after injury is fundamental.

Macrophages may be polarized in vitro depending on various stimuli, resulting in the subdivision of macrophages into two major and other intermediate populations 7. A common framework for macrophage‐activation nomenclature has been proposed 8. Pro‐inflammatory macrophages are generated in the presence of IFNγ + LPS and express high levels of inducible nitric oxide synthase (iNOS) 9 and CCR7 10, 11, whereas anti‐inflammatory macrophages, which are generated in the presence of IL4 + IL13 12, express arginase 1 (Arg1), and promote angiogenesis 7.

Pro‐inflammatory macrophages appear to accumulate after the onset of injury 13, 14, 15. They release cytokines that exacerbate the injury, amplify the inflammatory response and also contribute to myofibroblast proliferation and recruitment of fibrocytes 16, 17. Studies of multiple cytokine‐deficient mice have demonstrated that fibrosis is strongly linked with the development of a Th2‐biased response involving IL4, IL5, and IL13 18, 19, 20, 21. For instance, daily doses of IFNγ slow the fibrotic process 22, and Wynn et al. demonstrated that fibrosis reduction is associated with decreased production of IL4 and IL13 and an increased Th1 immune response 1. Moreover, Th2 cytokine production is observed when the acute phase of inflammation is finished, which in turn promotes polarization and recruitment of so‐called “pro‐resolving macrophages” 23. In parallel, apoptotic cells are recognized and phagocytosed by macrophages, an event that also promotes macrophage alternative activation 24. These cells are supposed to create an anti‐inflammatory environment and promote wound repair. When injury is persistent, pro‐resolving macrophages apparently assume an important pro‐fibrotic role, secreting large amounts of pro‐fibrotic factors such as TGF‐β 25. However, in some cases, pro‐resolving macrophages are associated with suppression rather than induction of fibrosis 26.

The present study sought to investigate the association between different macrophage subtypes and the progression of fibrosis using the unilateral ureteral obstruction (UUO) model of disease. The leading hypothesis is that a pro‐inflammatory rather than only a pro‐resolving milieu can induce fibrosis formation throughout the chronicity of the experimental model. Initially, we observed an increase in the pro‐inflammatory macrophage population (p40IL12^+^CCR7^+^CD11b^+^) during the initial stages of renal injury, whereas the number of pro‐resolving macrophages (Arg1^+^IL10^+^CD206^+^CD11b^+^) increased throughout the chronicity of disease. Then, we injected in vitro differentiated macrophages into macrophage‐depleted animals during the initial stage of the disease. Injection of M (IFNγ + LPS) instead of M (IL4 + IL13) or M0 led to accentuated ECM deposition, increased levels of anti‐inflammatory molecules and impaired organ function. Injection of M (IFNγ + LPS) unable to switch to a pro‐resolving phenotype worsened the fibrotic scenario in our model of disease. Accordingly, we observed that p40IL12^+^CCR7^+^CD11b^+^ macrophages at early stages create conditions for inflammatory milieu formation and consequent fibrosis development.

## Materials and Methods

### Animal studies

Six‐ to eight‐week‐old male Rag1^−/−^, Arg‐1 YFP, and CD45.1^+^ mice in a C57BL/6 background and control C57BL/6 mice and Stat6^−/−^ mice in a BALB/c background were housed in a pathogen‐free facility under a 12‐h light/dark cycle in a temperature‐controlled room at 21–23°C with free access to water and food. On day 0, the mice were anesthetized with ketamine‐xylazine (Agribrands, Sao Paulo, Brazil), and UUO was performed by complete left ureter ligation. The animals were placed in individual cages and warmed by indirect light until they had completely recovered from anesthesia. Seven days later, the mice were sacrificed for biochemical, histological, and molecular analyses. All procedures were approved by the ethics committee of the University of Sao Paulo (document 45/2009).

### Renal function parameters

Urinary protein/creatinine ratios were measured in samples collected from the pelvis of obstructed mice 7 days post‐surgery and from the bladder before surgery. All samples were analyzed by colorimetric assays using commercially available kits for creatinine and protein measurement (Labtest, Minas Gerais, Brazil). Normalization was achieved by dividing the protein/creatinine ratio at day 7 by the ratio before surgery in each group of animals.

### Sirius red staining

Kidneys were harvested and placed in 10% buffered formaldehyde for fixation. Slides were de‐paraffinized, rehydrated, and immersed in saturated picric acid solution for 15 min and then in Picrosirius (Abcam, Cambridge, United Kingdon) for 20 min. Counterstaining was performed using Harris hematoxylin. Picrosirius‐stained sections were analyzed by an Olympus BX50 microscope. Manual photographs were taken of the cortex, magnified at 20×, and observed under polarized light. Images of at least 20 different fields in each slide were obtained, and structures such as the glomeruli, subcapsular cortex, large vessels, and medulla were excluded. For morphometric analysis, image processing and analysis in Java Image J software (National Institutes of Health, Betesda, MD, USA) was used. The result of the analysis was represented by percentage (% staining/field) and refers to the proportion of the stained volume to the total cortical interstitial volume.

### Immunostaining

Immunostaining was performed on paraffin sections using a microwave‐based antigen retrieval technique. The antibodies used in this study included α‐sma (diluted 1:300; DAKO, Denmark) or a negative control reagent. Sections were incubated with labeled polymer (Dual Link System‐HRP, Glostrup, Denmark, DAKO). Staining was completed by incubating sections for 1–3 min with 3,3‐0‐diaminobenzidine substrate‐chromogen, resulting in the formation of a brown‐colored precipitate at the antigen site. Finally, hematoxylin counterstaining was performed.

### Quantitative real‐time PCR

RT‐PCR was performed using the Taqman real‐time PCR assay (Applied Biosystem, Foster City, CA, USA) for the following molecules: HPRT (Mm00446968_m1), IL‐1β (Mm00434228_m1), type 1 collagen (Mm00801666_g1), arginase 1 (Mm01190441_g1), TSLP (Mm00498739_m1), *tnf‐α* (Mm00443258_m1), iNOS (*nos2*) (Mm00440485_m1), TGF‐β (Mm03024053_m1), CD68 (Mm03047340_m1), and IRF5 (Mm00496477_m1). We also utilized some SYBR primers: CCR7: F‐GACAGCTATCCCCAAAACGACA, R‐GCCTCCGAAGACTACTCAACCA, IL‐10: F‐CCAAGCCTTATCGGAAATGA, and R‐TTTTCACAGGGGAGAAAT. Cycling conditions were as follow: 10 min at 95°C, followed by 45 cycles of 20 sec at 95°C, 20 sec at 58°C, and 20 sec at 72°C. Analysis was performed using Sequence Detection Software 1.9 (SDS, Gent, Belgium). mRNA expression was normalized to HPRT expression.

### Macrophage isolation procedures

Primary macrophages were obtained from bone marrow cells. Mice were anesthetized as described previously and euthanized by cervical dislocation, and their tibias and femurs were removed and washed with PBS. Cells were filtered through sterile polystyrene syringes (70–100 μm) and divided into 10 Petri dishes (100 × 20 mm; BD, Franklin Lakes) supplemented with 10 ml of medium containing 60% DMEM‐High (Invitrogen, Carlsberg, CA, USA), 25% L929 cell culture supernatant and 15% fetal bovine serum. On the 4th day of culture, we added an additional 10 ml of the same solution. We collected the cells on the 17th day using accutase (MP Biomedicals, CA) after washing with PBS. Macrophages were considered M0 when cultured with DMEM‐High (Invitrogen) and 5% fetal bovine serum for 24 h. A group of macrophages was cultured for 24 h in the presence of 10 ng/ml LPS (Sigma–Aldrich, MO, EUA) and 50 ηg/ml IFNγ (R&D Systems, MN, EUA) diluted in DMEM‐High (Invitrogen) and supplemented with 5% fetal bovine serum. Another group of macrophages was cultured in the presence of 10 ηg/ml IL4 (Peprotech, CA, EUA) and 10 ηg/ml IL13 (R&D Systems, MN, EUA) for 24 h under the same conditions described above. An alternative protocol for polarizing macrophages consisted of washing bone marrow cells with PBS, filtering them with polystyrene syringes (70–100 μm) and growing them in the presence of 10 ηg/ml M‐CSF or 10 ηg/ml GM‐CSF diluted in DMEM‐High (Invitrogen) supplemented with 5% fetal bovine serum for 7 days.

### Liposome preparation and macrophage depletion

Clodronate (Schering, São Paulo, Brazil) was entrapped in liposomes by ether injection. Typically, 0.5 ml of an ether solution containing 50 mg of phosphatidylcholine and 8 mg of cholesterol was injected (0.2 ml/min) into 5 ml of a 50 mmol/l clodronate aqueous solution maintained at 42°C. The ether solution was injected with a syringe adapted with a KD Scientific Inc. Model KDS120 Push‐Pull Pump equipped with a fine‐gauge needle (N° 3D). During injection, a nitrogen stream was bubbled into the clodronate solution, which was continued after liposome formation until removal of residual solvent. The liposome suspension was centrifuged at 22,800*g* for 30 min (Hitachi Himac CR20B2 centrifuge; Hitachi Ltd., Tokyo, Japan) at 25°C. The liposome‐containing pellet was washed twice by centrifugation under the same conditions in 0.9% (w/v) NaCl solution. The final pellet was resuspended in 2 ml of saline solution. The final phosphatidylcholine and clodronate concentrations in the liposomes were 10 mmol/l and 0.5 mmol/l, respectively. The yield of entrapped clodronate was approximately 1% of the initial quantity added. Mice were injected i.p. with 100 μl of liposome preparation (6 μg of clodronate) 24 h before UUO surgery. In addition, 200 μl of the liposome preparation (12 μg of clodronate) was administered i.p. during surgery.

### Flow cytometry

Kidneys were collected from sacrificed animals for flow cytometry analysis following the standard manufacturer's protocol. Briefly, kidneys were reperfused, excised, and incubated in collagenase IV for 30 min. Then, monocytes were separated by Percoll (Sigma, St. Louis) gradient. We analyzed the renal cells by multicolor flow cytometry. The monoclonal antibodies used were F4/80 PerCP, CD11b PE, CD206 FITC, MIG PE, IL10 APC, p40 (IL12/IL23) PE‐Cy7, CCR7 PerCP‐Cy5.5, IFNγ FITC, CD45.1 Pacific Blue, CD45.2 APC‐Cy7, GM‐CSF PE, MHC II IAB PerCP, and CD36 FITC (all purchased from BD Biosciences, Franklin Lakes). Samples were acquired on a FACSCanto using FACSDIVA software (BD Biosciences), followed by analysis with FLOWJO software (Tree Star, San Carlo, CA). Fluorescence voltages were determined using matched unstained cells. Compensation was performed using cells (BD Biosciences) single‐stained with CD3 PerCP, CD4 FITC, CD8 APC‐CY7, CD4 PE‐CY7, CD4 PerCP‐Cy5.5, CD3 PE, or CD3 APC. Samples were acquired up to at least 200,000 events in a live mononuclear gate, followed by a doublets exclusion gate, following the standard manufacturer's procedure. The compensation process was performed according to the “fluorescence minus one” method.

### Cytokine profile

Kidney cells were lysed in RIPA buffer with protease inhibitors. A Bio‐Plex Mouse Plex Cytokine Assay Kit (Bio‐Rad Laboratories, Inc., Hercules, CA) was used to test samples for the presence of 23 cytokines. The assay was read with a Bio‐Plex Suspension Array System, and the data were analyzed using Bio‐Plex Manager software version 4.0. Standard curves ranged from 32,000 to 1.95 pg/ml.

### Transwell migration assay system

Macrophages were placed in the top of Transwell membrane system (BD, CA) with pores with a diameter of 8 μ; 100 nM leukotriene B4 diluted in DMEM‐High (Invitrogen) supplemented with 5% fetal bovine serum was added to the bottom for 8 h. After this period, the membranes were removed, and the cells that were in the bottom were fixed with 10% buffered formalin and stained with hematoxylin. To quantify migrated cells, the membranes were observed under a light microscope (Zeiss, Oberkochen, Germany).

### Preparation of type I collagen and fibronectin three‐dimensional matrix (3D)

To prepare the three‐dimensional matrix, 212 ml (0.850 mg/ml) of type I collagen, 100 ml of 10× DMEM and 100 ml of 10× RB buffer were mixed in a tube. In another tube, 500 μl of PBS, 10 μl of FBS, 10 μl of fibronectin from human plasma and 10 μl of fluorescein‐conjugated DQ Type I Collagen (Life Technologies) were added. The tubes were centrifuged at 4°C for 3 min at 10,000 RPM. To adjust the pH to 7.4, 60 ml of HCl was added to the mixture, followed by thorough mixing by vortexing. From this preparation, 130 μl of gel was placed in culture plates containing a glass coverslip of 12 mm at the center. The disks were incubated at 37°C for 2 h to polymerize the gel bottom. Then, in a volume of 75 μl of gel, 2 × 10^3^ cells were added on the already polymerized matrix and incubated at 37°C for 2 h. Next, 2 ml of the respective culture medium was added. For the migration assay, the plates remained in the incubator for 16 h, sufficient for cells to acquire the elongated and cylindrical morphology typical of cells in 3‐D culture. After confirming that the cells were 3‐D, the medium was replaced with DMEM containing 10% (60 mM) HEPES buffer, and finally, a layer of mineral oil was added on the culture medium to prevent gas exchange during the experiment.

### ECM gene array

Total macrophage RNA was isolated using TRIzol reagent (Invitrogen) according to the manufacturer's instructions. A total of 1 µg was then reverse transcribed using the First Strand Synthesis Kit (Qiagen, Hilden, Germany) and subsequently loaded onto extracellular matrix and adhesion molecules RT profiler array according to the manufacturer's instructions (Qiagen). Qiagen's online Web analysis tool was used to produce comparative heat maps, and fold change was calculated by determining the ratio of mRNA levels to control values using the Δ threshold cycle (Ct) method (2^−ΔΔCt^). All data were normalized to an average of five housekeeping genes, Gusb, Hprt, Hsp90ab1, Gapdh, and Actb. PCR conditions used: hold for 10 min at 95°C, followed by 45 cycles of 15 sec at 95°C and 60 sec at 60°C.

### Bioinformatics analysis

The common and exclusive differentially expressed genes across the comparisons were identified using the Gene List Venn Diagram software (Gent, Belgium) 27. Then, subsets of common and exclusive genes were organized into diagrams to categorize the molecular changes related to each specific macrophage population. Next, we gathered information regarding substrate and protein–protein interactions among the differentially expressed genes and other interacting proteins to delineate the proteolytic profiles associated with each macrophage subtype. The protease‐substrate data were obtained from the PMAP 28 and CutDB 29 databases, which compile information regarding the substrate recognition specificity of proteases, as well as from a review that assembled information about the known substrates of metalloproteinases (MMPs) 30. In parallel, protein–protein interaction data were obtained from the STRING database 31, which gathers information about known and predicted protein interactions. We only used the “experiments” evidence type as an active prediction method to identify protein–protein interactions in STRING. With this interaction profile data, proteolytic networks were constructed to analyze proteolytic profiles and pathways specific to each macrophage subtype. Networks were built using Cytoscape software (National Institutes of Health, Betesda, MD, USA) 32, which allows network editing for improved visualization of the molecular components in networks. Nodes representing differentially expressed genes up‐ and down‐regulated in each condition are colored in red and green, respectively. In addition to DE genes, nodes with three or more connections are highlighted with a yellow border to facilitate visualization.

### Statistics

The data are presented as the mean ± SEM. Differences between groups were identified using ANOVA (with Tukey's post test) and Student's *t* tests. Differences were considered significant when the *P*‐value was <0.05. All statistical analyses were performed using GraphPad Prism (La Jolla, CA, USA).

## Results

### Distinct macrophage subtypes at early and late days after injury

**Figure 1:**
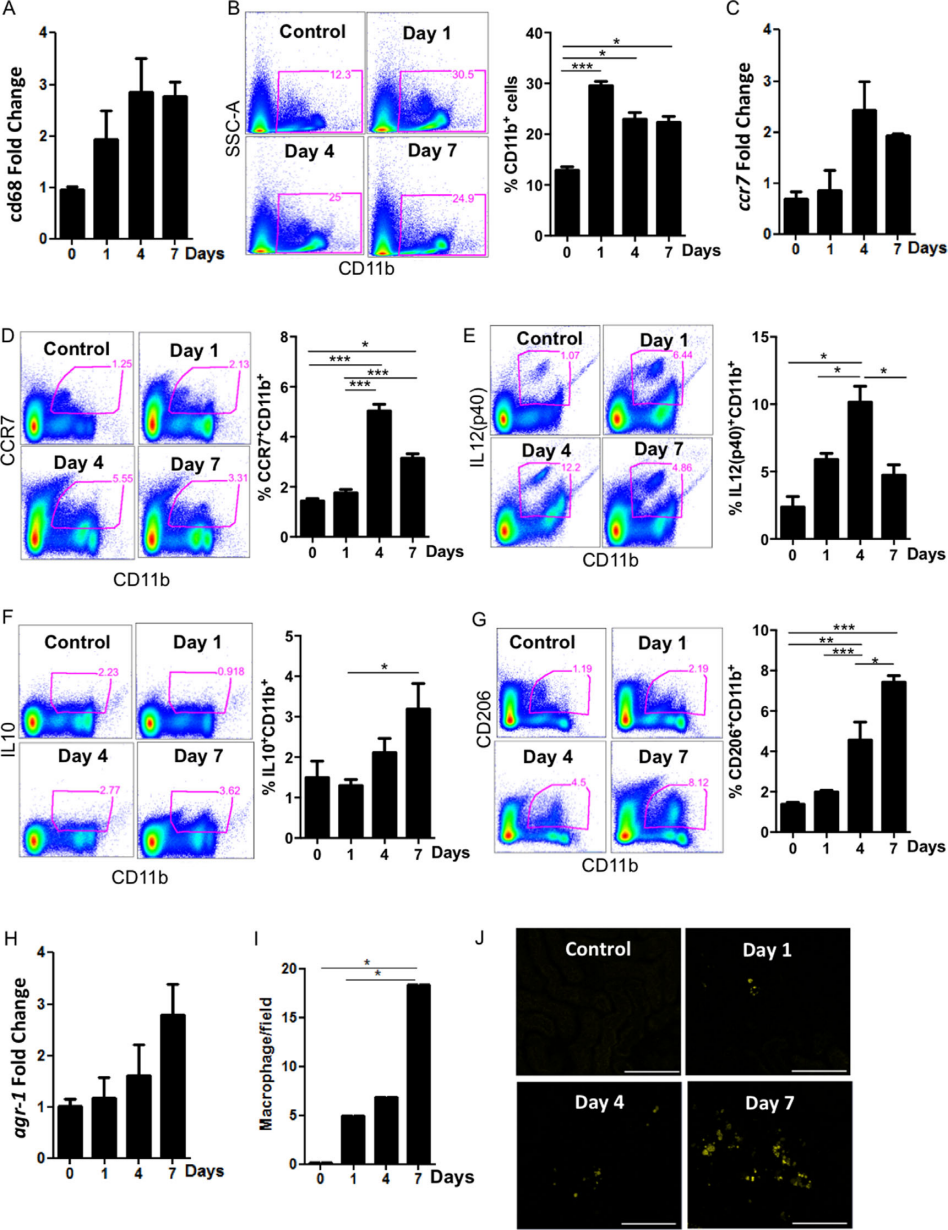
Time course of the macrophage profile during UUO. Kidneys of WT mice were analyzed before (0) and at 1, 4, and 7 days after ureteral obstruction. Macrophages were quantified in (A) and (B) by cd68 mRNA expression and CD11b^+^ cell infiltration, respectively. The mRNA expression (C) and protein expression (D) of the pro‐inflammatory marker CCR7 were analyzed. E: The percentage of kidney p40(IL12/23)^+^CD11b^+^ cells was determined. The pro‐resolving markers (F) IL10^+^ and (G) CD206^+^ were analyzed by flow cytometry. H: Arg1 mRNA expression was quantified by real‐time PCR. I and J: Representative photomicrographs and quantification of Arg1 YFP cells by confocal microscopy before ureteral obstruction (0) and at 1, 4, and 7 days after surgery. qPCR data were normalized to HPRT expression, and the mean expression in WT non‐obstructed control mice was set to 1. In J, the bars represent 100 μm in all photomicrographs. *P < 0.05; **P < 0.01; and ***P < 0.001. *n* = 5 animals per group in three different experiments.

Macrophages play an essential role in the development of fibrosis after sustained tissue injury 21, 33, 34. Here, we observed the dynamics of macrophage subtypes in an established model of renal chronic injury. We observed an early increase in CD68 mRNA in obstructed kidneys (Fig. 1A), accompanied by increased CD11b^+^, gated on CD45^+^ live cells after doublets exclusion, immediately after surgery (Fig. 1B). Moreover, most tissue macrophages exhibited a pro‐inflammatory phenotype that peaked at day 4, with high levels of CCR7 (Fig. 1C and D) and increased p40 (IL12/IL23) protein expression (Fig. 1E) compared to non‐obstructed control mice (day 0). Conversely, tissue macrophages mainly presented a pro‐resolving phenotype throughout the progression of the model, exhibiting decreased levels of IL10 expression early after surgery and IL10 levels comparable to non‐obstructed mice at day 7 (Fig. 1F). We also observed high levels of CD206 protein expression at day 7 compared to previous time periods and the control mice (Fig. 1G). Moreover, *Arg1* expression increased with the chronicity of the disease (Fig. 1H). These results were corroborated by the increased number of interstitial Arg1^+/+^ cells at day 7 compared to the initial stages of the disease and to control mice (Fig. 1I and J). Taken together, these data indicate that pro‐inflammatory macrophages are present in injured organs during the initial stages, whereas the population of pro‐resolving macrophages increases during the chronic phase of renal disease.

### Pro‐inflammatory macrophages accentuate kidney fibrosis

**Figure 2:**
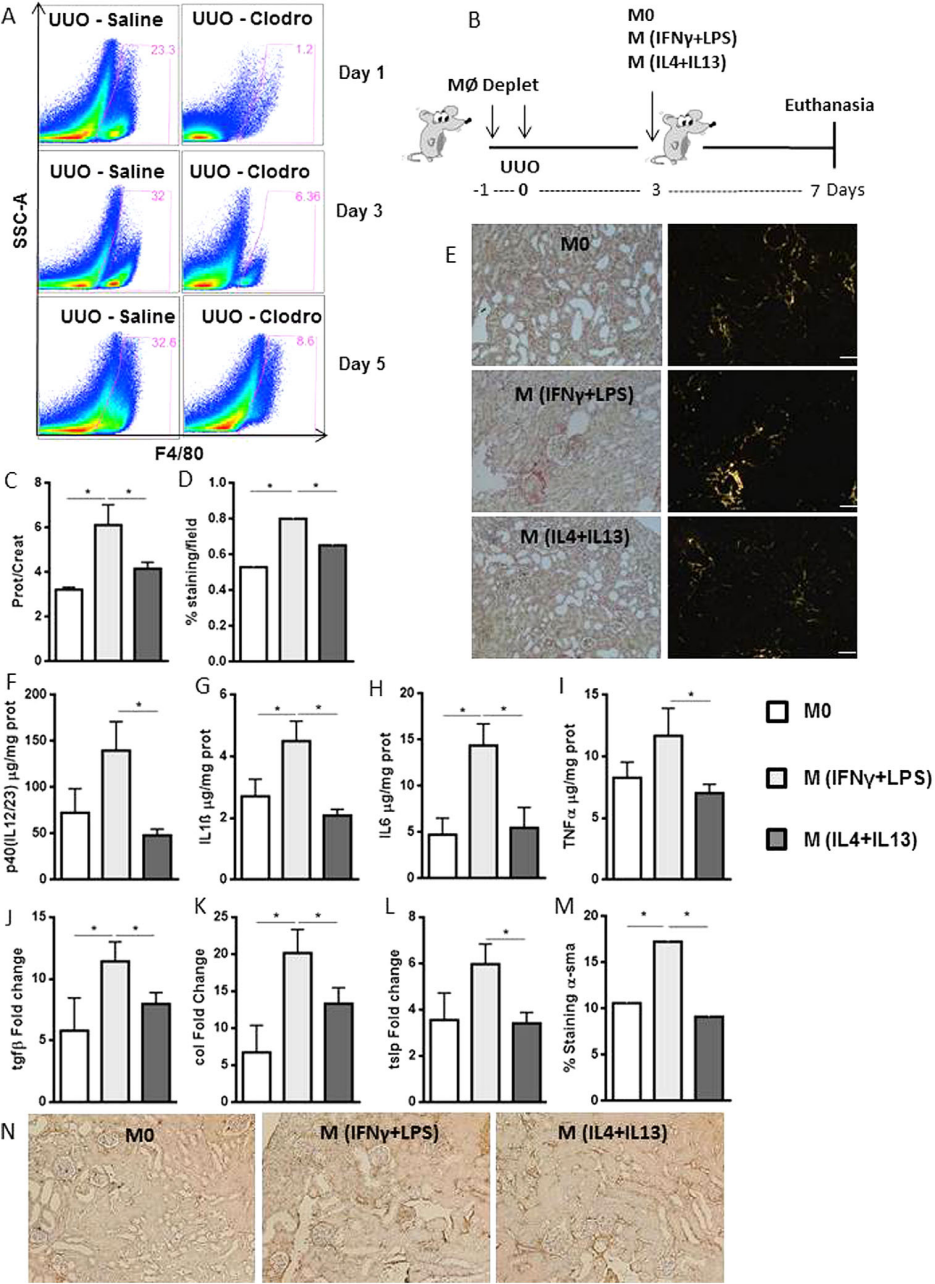
M (IFNγ + LPS) macrophage injection accentuates inflammation and fibrosis compared with M0 and M (IL4 + IL13) injection. A: Time course of macrophage depletion with clodronate‐encapsulated liposomes or saline control. Kidney F4/80^+^ cells were analyzed by flow cytometry at days 1, 3, and 5 after obstruction. B: Schematic representation of the experimental design. Macrophages of Rag1^−/−^ animals were depleted 1 day before and during ureteral obstruction surgery. A total of 5 × 10^4^ M0, M (IFNγ + LPS), or M (IL4 + IL13) macrophages were injected intravenously on the 3rd day after UUO, and the mice were euthanized on the seventh day. C: Proteinuria in the obstructed pelvises was normalized to that in the urine of the animals before surgery. D and E: Quantification and representative photomicrographs of collagen deposition, as analyzed by Sirius Red staining, in the kidneys of control Rag1^−/−^ macrophage‐depleted animals following injection. Animals were analyzed for (F) p40(IL12/23), (G) IL1β, (H) IL6 and (I) TNFα protein levels using Luminex technology and for (J) tgfβ, (K) type I collagen and (L) tslp mRNA expression levels on day 7 after UUO. M and N: Quantification and representative photomicrographs of α‐sma deposition, as analyzed by immunohistochemistry. In E and N, the bars represent 50 μm in all photomicrographs. Sirius Red staining and α‐sma deposition are represented by the percentage of stained area out of the total area in the field. qPCR data were normalized to HPRT expression, and the mean expression in the Rag1^−/−^ control mice was considered 1. *P < 0.05; and ***P < 0.001. *n* = 5 animals per group in three different experiments. α‐sma, α‐small muscle actin.

We further investigated which cell subpopulation is the main inducer of fibrosis: the pro‐inflammatory macrophages observed in the initial phase or the pro‐resolving macrophages observed during the fibrotic phase. We initially depleted macrophages of Rag1^−/−^ mice submitted to UUO using liposome‐encapsulated clodronate (Fig. 2A). Three days after ureteral obstruction, we intravenously transferred M0, M (IFNγ + LPS) or M (IL4 + IL13) and assessed the degree of inflammation and fibrosis 4 days later, as displayed in the experimental design (Fig. 2B). Animals injected with M (IFNγ + LPS) exhibited higher proteinuria (Fig. 2C) and increased collagen deposition at day 7 (Fig. 2D and E) compared to mice injected with M0 or M (IL4 + IL13) cells, in contrast to previous data supporting a pro‐fibrotic role of these cells 35, 36.

Moreover, animals injected with M (IFNγ + LPS) exhibited increased production of kidney p40IL12, IL1β, IL6, and TNFα on the 7th day after UUO (Fig. 2F–I). Unexpectedly, in addition to inflammatory components, these animals exhibited increased gene expression of *tgfβ*, *collagen1*, and *tslp*, which are all related to fibrosis (Fig. 2J–L). Furthermore, α‐SMA deposition was higher after M (IFNγ + LPS) injection compared with M0 and M (IL4 + IL13) injection (Fig. 2M and N). Therefore, injection of M (IFNγ + LPS) led to the perpetuation of an inflammatory milieu, which ultimately favored the pro‐fibrotic environment observed during the chronic phase of obstructive kidney disease.

Because the macrophages at the early stages of UUO expressed CCR7 and, later, CD206, we reasoned that the injection of sorted CCR7^+^CD11b^+^ or CD206^+^CD11b^+^ macrophages would generate the same effects observed before cell sorting. We either sorted cells after in vitro differentiation or sorted CCR7^+^CD11b^+^ and CD206^+^CD11b^+^ cells from congenic mice (CD45.1^+^) at days 3 and 7 after UUO, respectively. CCR7^+^CD11b^+^ injection into macrophage‐depleted mice led to increased proteinuria (Sup. Fig. S1A and G) and increased collagen deposition (Sup. Fig. S1B and H). Although we observed higher expression of p40IL12/23 following injection of CCR7^+^CD11b^+^ derived from in vitro differentiation (Sup. Fig. S1C), there were no differences in IL1β, IFNγ, and TNFα production (Sup. Fig. S1D–F). By contrast, the injection of CCR7^+^CD11b^+^ cells derived from congenic mice revealed increased production of IFNγ and TNFα, although there were no differences in p40IL12/23 and IL1β (Sup. Fig. S1I–L) expression. These data suggest that pro‐inflammatory CCR7^+^CD11b^+^ macrophage injection increases fibrosis formation compared with the injection of pro‐resolving macrophages.

We also compared the development of fibrosis after injection of in vitro‐differentiated M (GMCSF) or M (MCSF). Initial characterization of these cells revealed that M (GMCSF) had spiculated morphology, whereas M (MCSF) was fibroblastoid (Sup. Fig. S2A). M (GMCSF) constitutively expressed higher levels of CCR7, GMCSF, class II MHC and p40IL12/23proteins (Sup. Fig. S2B) in addition to higher levels of CCR7, iNOS, IRF5, and IL1βmRNA (Sup. Fig. S2C) compared with M (MCSF). By contrast, M (MCSF) expressed higher levels of CD206 and CD36 proteins (Sup. Fig. S2B) and IL10 mRNA (Sup. Fig. S2C). The expression levels of the remaining evaluated proteins, including F4/80 and IFNγ (Sup. Fig. S2B), and CD68, TNFα and Arg‐1 mRNA (Sup. Fig. S2C) were similar in M (GMCSF) and M (MCSF) macrophages.

Injection of 5 × 10^4^ cells did not alter proteinuria and collagen deposition when we compared injection of both macrophages (Sup. Fig. S2D–F). Injection of 1 × 10^6^ M (GMCSF) macrophages led to a worse fibrotic environment compared with injection of 1 × 10^6^ M (MCSF). We observed that 1 × 10^6^ M (GMCSF) led to increases in proteinuria (Sup. Fig. S2G), type 1 collagen mRNA expression and collagen deposition (Sup. Fig. S2H and I) compared to 1 × 10^6^ M (MCSF). Again, our results suggest that fibrosis formation is related to the injection of pro‐inflammatory rather than pro‐resolving macrophages.

To clarify whether injected macrophages exert their function in a paracrine or endocrine manner, we next investigated whether the injected differentiated macrophages reach the injured kidneys. For this purpose, we injected CD45.1^+^macrophages: M0, M (IFNγ + LPS) or M (IL4 + IL13). Although approximately 40% of the macrophages reaching the obstructed kidneys (Sup. Fig. S3A), the percentage of kidney CD11b^+^ cells expressing CD45.1^+^ cells was approximately 3% (Sup. Fig. S3B). This percentage represents approximately 1 × 10^2^ of 5 × 10^4^ injected cells for all macrophage subtypes injected (Sup. Fig. S3C). We also derived M (IFNγ + LPS) or M (IL4 + IL13) from GFP^+^ mice and injected them using the same experimental design (Sup. Fig. S3D). We did not detect CD45^+^ cells in spleen and lung of the injected animals (Sup. Fig. S3E and F). By contrast, the majority of infused cells reached the liver (Sup. Fig. S3G), representing approximately 1 × 10^4^ of 5 × 10^4^ injected cells for M (IFNγ + LPS) or M (IL4 + IL13) conditions (Sup. Fig. S3H). These data suggest that only a portion of the macrophages in the obstructed kidneys were derived from the injected cells. The remaining kidney macrophages were probably derived from blood circulation or even from tissue proliferation, as inferred by different authors 37, 38.

These results indicate that the injected macrophages exert their effects in an endocrine manner. These endocrine effects were evaluated by analyzing cytokines in the sera of the obstructed animals. M (IFNγ + LPS) injection led to increased IL6, IL10 and IL13 protein production compared to M0, M (IL4 + IL13) injection (Sup. Fig. S3I–K). Altogether these data indicate that M (IFNγ + LPS) macrophages are more prone to induce fibrosis compared to M (IL4 + IL13) macrophages, the former cells generate an intense pro‐inflammatory milieu, even if they cannot reach the injured organ.

### Pro‐inflammatory macrophages generated from Stat6^−/−^ mice enhance fibrosis

To clarify whether the development of renal fibrosis is associated with the mere presence of pro‐inflammatory macrophages in the early damage response or with the change in phenotype of the injected macrophages over time, we injected M (IFNγ + LPS) that were unable to change into CD206^+^CD11b^+^ macrophages. We took advantage of pro‐inflammatory macrophages derived from Stat6^−/−^ animals because Stat6 participates in the IL4 and IL13 signaling pathways 39, 40, 41.

Initially, we characterized in vitro differentiated M (IFNγ + LPS) and M (IL4 + IL13) cells and compared then to macrophages generated from Stat6^−/−^ mice. Compared to control macrophages (Mo), M (IFNγ + LPS) exhibited a spiculated morphology and increased protein levels of CCR7, GMCSF, MHC II, and p40IL12/23, in contrast to M (IL4 + IL13), which presented fibroblastoid morphology and increased protein levels of CD206 (Sup. Fig. S4A–G). These data corroborate previous data concerning macrophage biology 7, 42. In addition, compared to macrophages derived from WT mice, Stat6^−/−^ macrophages exhibited a pro‐inflammatory profile at baseline and also after exposure to different stimuli, including LPS + IFNγ, IL4 + IL13, and MCSF. We used MCSF because its signaling is independent of Stat6 and may thus lead to polarization toward an anti‐inflammatory profile. Stat6^−/−^ macrophages exhibited increased levels of CCR7, MIG (CXCL9) and p40 (IL12/23) and a reduced level of CD206 (Sup. Fig. S4H). Moreover, the addition of IL4 + IL13 or M‐CSF did not increase CD206 expression in bone marrow‐derived macrophages from Stat6^−/−^ animals.

**Figure 3:**
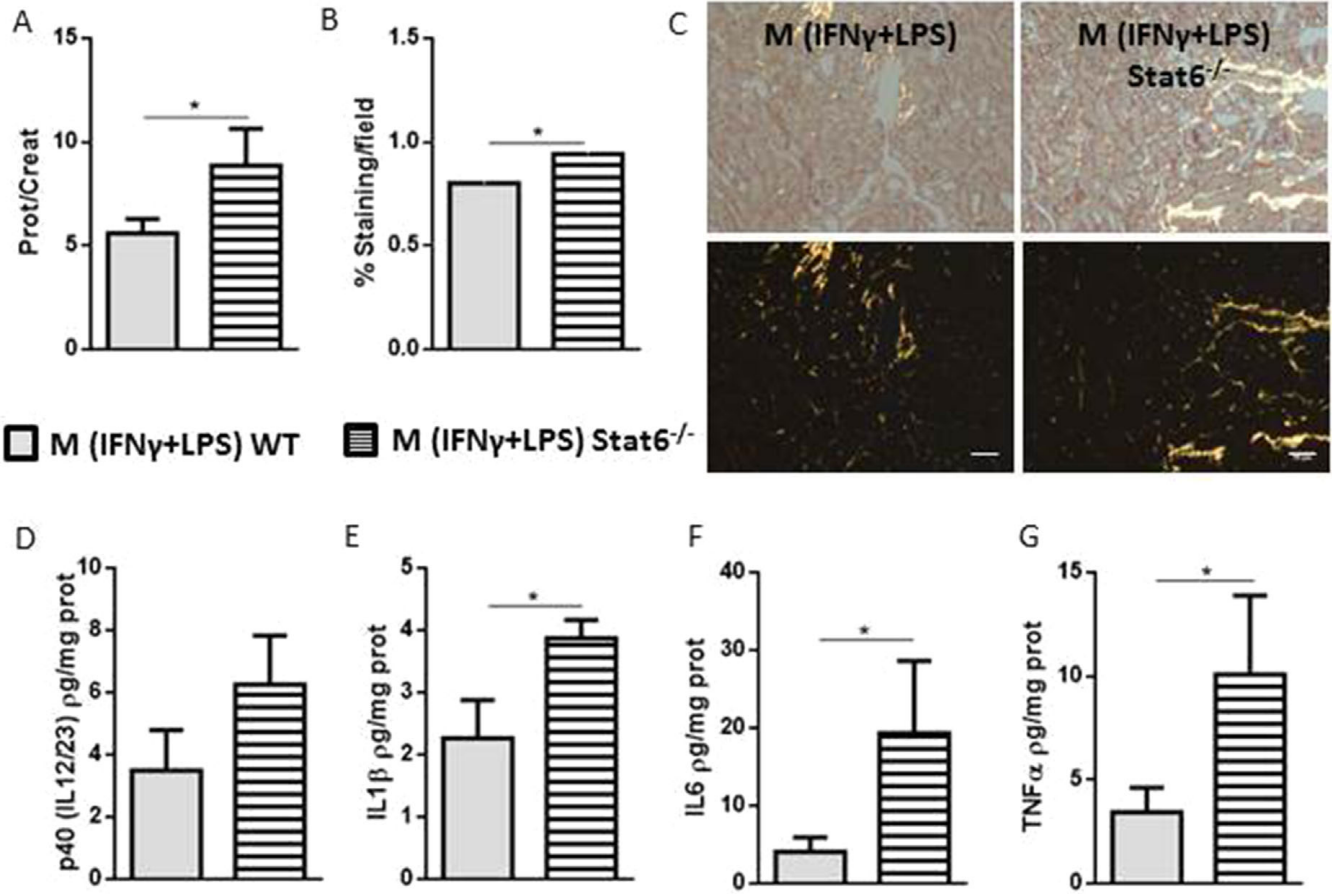
M (IFNγ + LPS) Stat6^−/−^ macrophage injection accentuates obstructive kidney fibrosis compared with M (IFNγ + LPS) Stat6^+/+^. Rag1^−/−^ animals were depleted of macrophages, and a total of 5 × 10^4^ M (IFNγ + LPS) Stat6^+/+^ or M (IFNγ + LPS) Stat6^−/−^ macrophages were injected intravenously on the 3rd day after UUO. The mice were euthanized on day 7. A: Proteinuria in the pelvises of the animals undergoing UUO was normalized to that in the urine of the animals before surgery. B and C: Quantification and representative photomicrographs of collagen deposition, as analyzed by Sirius Red staining, in the kidneys of macrophage‐injected animals. Kidney (D) p40 (IL12/23), (E) IL1β, (F) IL6 and (G) TNFα protein levels of the macrophage‐injected animals were analyzed using Luminex technology. In C, the white bars represent 50 μm in all photomicrographs. Sirius Red staining is represented by the percentage of the stained area out of the total area in the field. *P < 0.05; **P < 0.01; and ***P < 0.001. *n* = 5 animals per group in three different experiments.

We then transferred in vitro differentiated M (IFNγ + LPS) from either WT or Stat6^−/−^ mice into Rag1^−/−^macrophage‐depleted animals. Notably, injection of Stat6^−/−^ M (IFNγ + LPS) animals led to increased proteinuria (Fig. 3A) and collagen deposition in the kidneys of recipient animals compared to mice that received M (IFNγ + LPS) Stat6^+/+^ (Fig. 3B and C). Despite the lack of differences in p40IL12/23, we observed an increase in IL1β, IL6 and TNFα protein expression after Stat6^−/−^ M (IFNγ + LPS) injection (Fig. 3D–G). Taken together, these results support that the persistence of an inflammatory milieu throughout the injury derived from macrophage subsets (CCR7^+^CD11b^+^) correlates with worse renal parameters.

### CCR7^+^CD11b^+^ cells exhibit higher gene expression of inflammation/fibrosis‐related molecules

**Figure 4:**
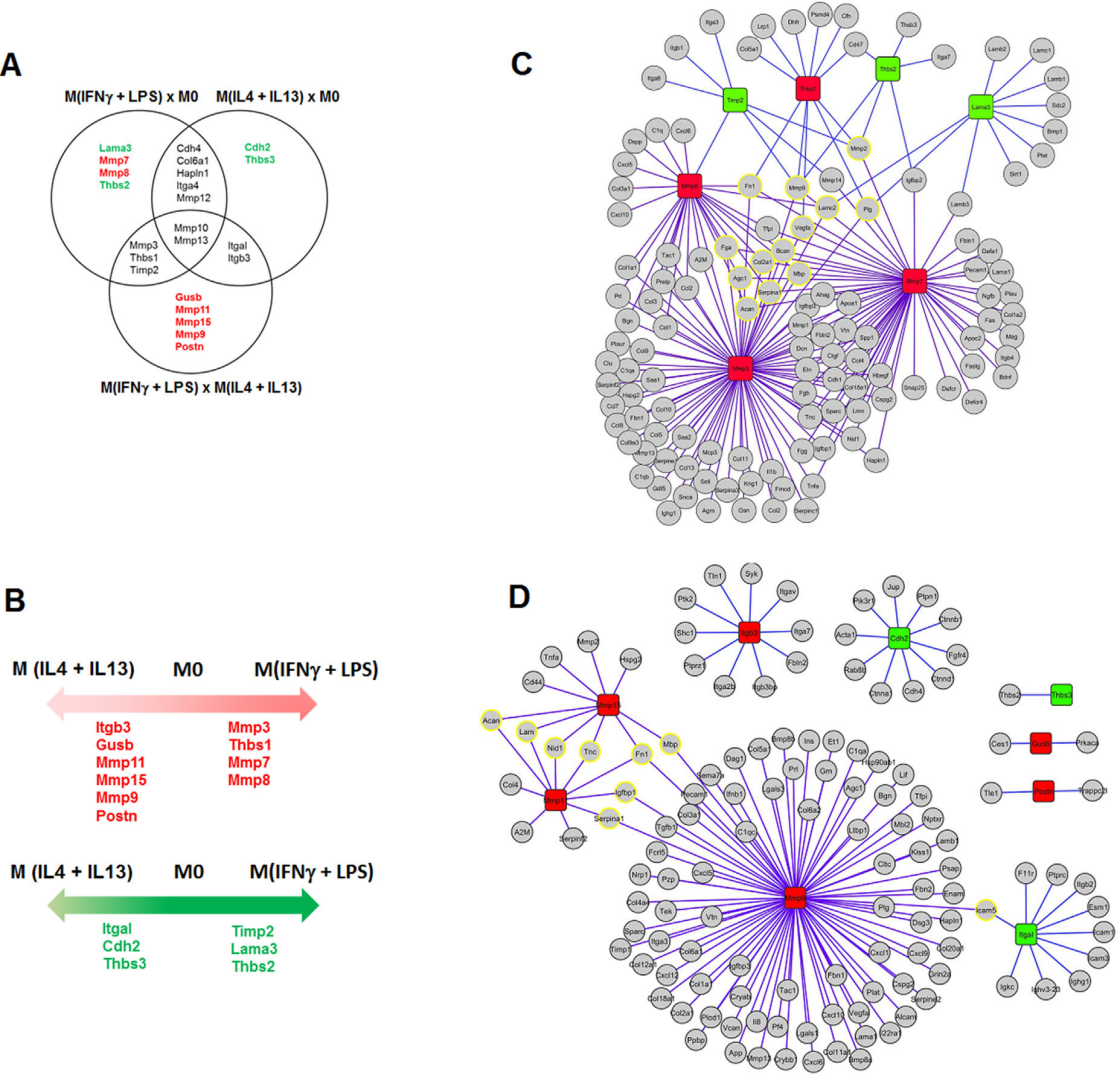
M (IFNγ + LPS) macrophages have high expression levels of inflammation/fibrosis‐related ECM molecules. PCR array to determine the expression of ECM‐related genes. A: Venn diagram showing gene expression changes identified by comparing the three different macrophage subpopulations. B: Common and unique genes identified in the comparisons in A. C–E: Network analysis of proteolytic pathways related to each macrophage phenotype. The purple lines represent enzyme‐substrate interactions, and the orange lines represent physical interactions. C: Comparisons between M0 and M (IFNγ + LPS) or M (IL4 + IL13) macrophages. The red boxes represent up‐regulated genes, and the green boxes depict down‐regulated genes in M0. D: Comparison between M (IFNγ + LPS) and M0 macrophages. The red boxes represent up‐regulated genes, and the green boxes depict down‐regulated genes in M (IFNγ + LPS). E: Comparison between M (IL4 + IL13) and M0 macrophages. The red boxes represent up‐regulated genes, and the green boxes depict down‐regulated genes in M (IL4 + IL13). All array data are presented as the average gene expression level of ten combined mice per group.

Because fibrosis development is directly related to ECM remodeling, we identified the expression patterns of 84 genes involved in ECM in each macrophage phenotype. Twenty‐seven genes were identified as differentially expressed in the comparisons among macrophage subtypes (Sup. Fig. S5A). Pairs of different macrophage subtypes were also compared. We investigated the differential expression profiles to determine the molecular changes associated with each macrophage subtype. For this purpose, a Venn diagram was constructed to indicate common and unique gene expression changes across the comparisons (Fig. 4A). Then, we organized the common and unique results identified in each comparison into diagrams to categorize molecular changes specifically related to each macrophage population (Fig. 4B).

We observed that M (IFNγ + LPS) expressed higher levels of thrombospondin‐1 (Thbs1) compared with M0 and M (IL4 + IL13). This protein is an important molecule that was previously associated with tubule‐interstitial fibrosis 43. We also observed that M (IFNγ + LPS) exhibited up‐regulation of MMP13, an MMP responsible for cleaving pro‐TNFα to bioactive TNFα 44. In addition to these two genes, M (IFNγ + LPS) also expressed higher levels of MMP3, MMP10, and MMP7 than M0 and M (IL4 + IL13). These MMPs are associated with innate immunity, wound healing and fibrosis 45, 46, 47. Therefore, we concluded that M (IFNγ + LPS) exhibit an ECM‐related gene expression profile and are involved in inflammation and fibrosis.

Most of the differentially expressed genes in the comparisons were associated with MMPs or inhibitors of MMPs. Because MMPs do not function alone but as part of proteolytic networks 48, we used an integrative approach to determine the MMP proteolytic networks for each macrophage subtype. We constructed networks using information from substrate and protein–protein interactions to reveal which genes were more central in the networks. We also aimed to reveal which ECM substrates were more likely to be regulated by differentially expressed MMPs in the proteolytic networks.

The analysis of proteolytic networks related to these molecular changes demonstrated that the M (IFNγ + LPS) network is mainly involved in the up‐regulation of Thbs1, Mmp7, Mmp8, and Mmp13 (Fig. 4C), whereas the M (IL4 + IL13) network (Fig. 4D) is organized around the up‐regulation of Itgb3, Mmp9, Mmp11, and Mmp15. MMP9 is a specific marker of M (IL4 + IL13), corroborating its relevance to the proteolytic pathways activated by these cells. Analogously, the most central substrates in the M (IFNγ + LPS) and M (IL4 + IL13) networks were Acan, Serpina1, Agc1, Fga, Col2a1, Mbp, Bcan, Vegfa, Fn1, Mmp9, Lamc2, Plg and Mmp2; and Acan, Lam, Nid1, Tnc, Serpina1, Igfbp1, Fn1, Mbp, and Icam5, respectively. These substrates may be degraded and therefore may be involved in the regulation of either macrophages or injured tissue biology. M (IFNγ + LPS) and M (IL4 + IL13) are related to different ECM‐degrading properties: M (IFNγ + LPS) cells are associated with a pro‐inflammatory and pro‐resolving phenotype 49.

Ultimately, we searched for differences in ECM remodeling among different macrophage subtypes. Despite producing similar amounts of active TGFβ (Sup. Fig. S5B), the differentiated macrophages exhibited decreased migration ability compared to M0 macrophages (Sup. Fig. S5C). In addition, M0 cells exhibited greater degradation of the ECM and thus migration through fibrotic tissue, as demonstrated by the type I and IV collagen degradation assays (Sup. Fig. S5D and E). This effect is attributable to the reduced differentiation of M0 cells compared to M (IFNγ + LPS) and M (IL4 + IL13). Although M (IFNγ + LPS) cells induced the up‐regulation of inflammatory and fibrosis‐related molecules, our results suggest that these cells do not produce higher levels of TGFβ or migrate more.

In summary, these data provide evidence that p40IL12^+^CCR7^+^CD11b^+^ macrophages are critical cells in fibrosis and that the fibrosis observed at late stages is directly proportional to the inflammation observed at early stages rather than attributable to the mere presence of Arg1^+^IL10^+^CD206^+^CD11b^+^ cells.

## Discussion

Tissue macrophage infiltration is a prominent feature associated with the severity of injury 50. Depleting phagocytes with liposome‐encapsulated clodronate reduces kidney damage in various renal injury models 5, 33, 51. However, recent studies have suggested that the phenotypes of recruited macrophages, rather than solely their presence, determine the extent of injury in renal parenchyma 51, 52. Consistent with this suggestion, Wang and colleagues have demonstrated that inflammatory macrophage infusion induces deterioration of renal function parameters in a pro‐inflammatory renal disease model 50. Moreover, the presence of pro‐inflammatory macrophages in injured organs increases the generation of ROS and induces tubular cell death 53. In addition, Lee et al. have demonstrated that injection of inflammatory macrophages into WT animals increases resolute markers after contact with proximal tubule cells and decreases iNOS expression over time 54. Furthermore, the pro‐resolving macrophage population increases along with the proliferation of tubular cells, particularly in chronic models of renal injury.

We observed that pro‐resolving macrophages, which are frequently considered directly responsible for fibrosis 55, 56, 57, 58, were the most abundant cell population at 7 days after UUO. We then injected M0, M (IFNγ + LPS) or M (IL4 + IL13) macrophages into animals undergoing UUO to evaluate the differential abilities of these macrophages to induce fibrosis. This same strategy of depletion and subsequent injection of macrophages was previously employed by Lee and colleagues 54 in a model of ischemia and reperfusion. Moreover, we used Rag1^−/−^animals in the current study to minimize any phenotypic changes of macrophages subsequent to lymphocyte activation. As expected, injection of M (IFNγ + LPS) macrophages led to an increase in the production of inflammatory molecules. Surprisingly, M (IFNγ + LPS) macrophage injection also led to greater collagen deposition and increased expression of pro‐fibrotic molecules such asα‐SMA, TGFβ, type‐1 collagen and TSLP. M (IL4 + IL13) macrophages may have a regulatory role in the context of fibrosis, as also hypothesized by Pesce et al. 26, 59, who have indicated that Arg1^+^ and Fizz1^+^ macrophages are suppressors rather than inducers of Th2 inflammation and fibrosis.

M2 macrophages produce Arg1, whereas M1 macrophages express iNOS. Both enzymes cleave L‐arginine; however, only Arg1 promotes the production of L‐proline, the primary amino acid component of collagen protein. Thus, M (IL4 + IL13) macrophages are considered responsible for the fibrotic process 58. However, our data indicate that M (IL4 + IL13) macrophages do not produce more TGFβ or migrate farther in vitro. Furthermore, injection of M (IL4 + IL13) macrophages into mice after UUO did not increase collagen synthesis to the same extent as that observed following injection of M (IFNγ + LPS) cells. Moreover, mice injected with M (IFNγ + LPS) macrophages unable to change their phenotype to that of pro‐resolving ones following IL4/IL13 signaling (Stat6^−/−^ macrophages) 39, 40, 60 exhibited worsening of the fibrotic process, confirming our data indicating that the presence of pro‐inflammatory macrophages precludes subsequent development of fibrosis.

After polarizing macrophages in vitro, we compared their ECM‐related gene expression profiles to determine the proteolytic pathways activated by each macrophage subtype. M (IFNγ + LPS) macrophages exhibited higher gene expression levels of MMP3 and MMP10 compared with M0 and M (IL4 + IL13) macrophages, in accordance with previous reports 61. M (IFNγ + LPS) macrophages also exhibited the highest expression of Thbs1, an ECM glycoprotein that bridges cell–cell interactions. Recombinant Thbs1 treatment has been shown to stimulate TNFα expression in bone marrow‐derived macrophages in time‐ and dose‐dependent manners 62. Interestingly, Thbs1^−/−^ mice exhibit defective macrophage IL10 production during the resolution phase of inflammation 63, suggesting that Thbs1 is involved in the chronicity of inflammation. Additionally, inhibition of Thbs1 mRNA expression prevents tubule‐interstitial fibrosis in a UUO model 43.

Overall, kidney damage caused by ureter obstruction is mediated by different components of innate immunity. This damage is chemotactic for different immune cells. Inflammatory macrophages, which are the first subtype to reach the kidney, generate renal damage that ultimately leads to fibrosis, whereas pro‐resolving macrophages appear during the chronic phase to control initial inflammation and injury. This damage occurs following the generation of an inflammatory milieu, which may worsen the repair process and exacerbate fibrosis. Further studies should be conducted to clarify the extent to which the presence of pro‐resolving macrophages in the kidney is protective against fibrosis formation. Additional investigations are also required to clarify the extent to which different macrophage subpopulations are attracted to the kidney and to examine the plasticity of these macrophages once they have infiltrated the kidney.

## Author Contributions

TTB performed the experiments, analyzed the results, and drafted the manuscript. MC and RCS assisted with surgery. HA and CAMF performed gene expression analysis. MCC performed confocal microscopy analysis. MIH supported the experiments by organizing the reagents and required materials. MESA and MFS assisted with the 3D culture system. KRP and IMC prepared liposome‐encapsulated clodronate. NOSC helped formulate and coordinate the study and edit the manuscript. All authors read and approved the final manuscript.

## Conflicts of Interest

The authors have no conflicts of interest to declare.

## Supporting information

Additional supporting information may be found in the online version of this article at the publisher's web‐site.

**Figure S1**. In vitro and ex vivo sorted CCR7^+^CD11b^+^ cell injection accentuates obstructive kidney fibrosis compared with CD206^+^CD11b^+^ cell injection.
**Figure S2**. M (GMCSF) macrophage injection accentuates obstructive kidney fibrosis compared with M (MCSF) injection.
**Figure S3**. M (IFNγ + LPS) macrophages induce inflammation and fibrosis in an endocrine manner.
**Figure S4**. M0, M (IFNγ + LPS) and M (IL4 + IL13) characterization.
**Figure S5**. M (IFNγ + LPS) and M (IL4 + IL13) macrophages present decreased migratory ability.
