# Supplementary figures and images for: Early infiltration of p40IL12+CCR7+CD11b+ cells is critical for fibrosis development
Source: Immun Inflamm Dis. 2016 Jul 21;4(3):300–14. doi: 10.1002/iid3.114 (PMC5004285; doi:10.1002/iid3.114)

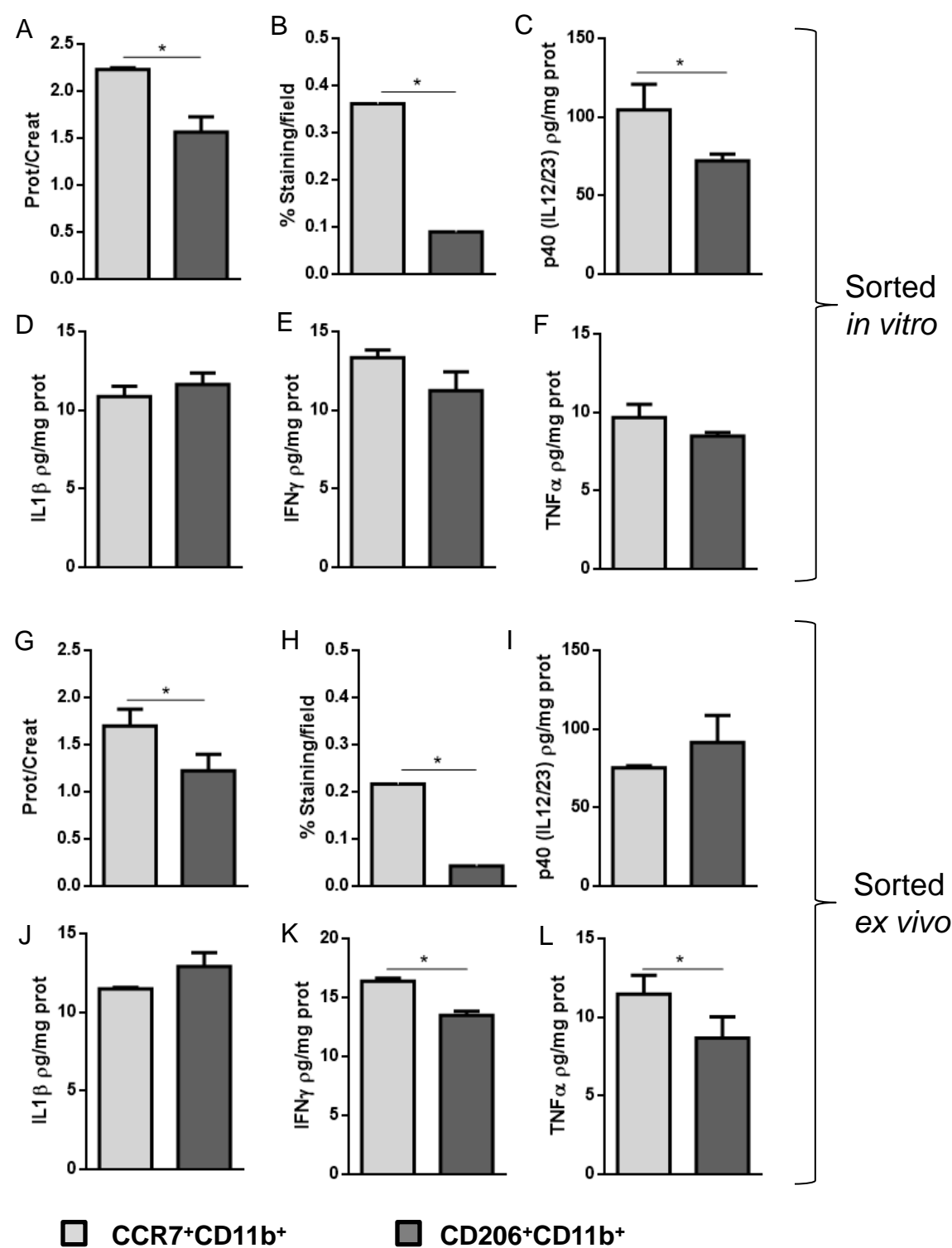

Sup. Fig. 1

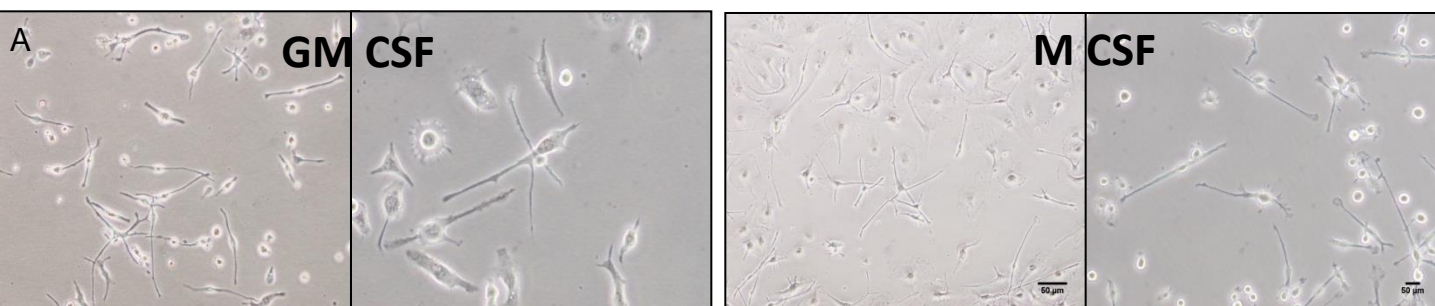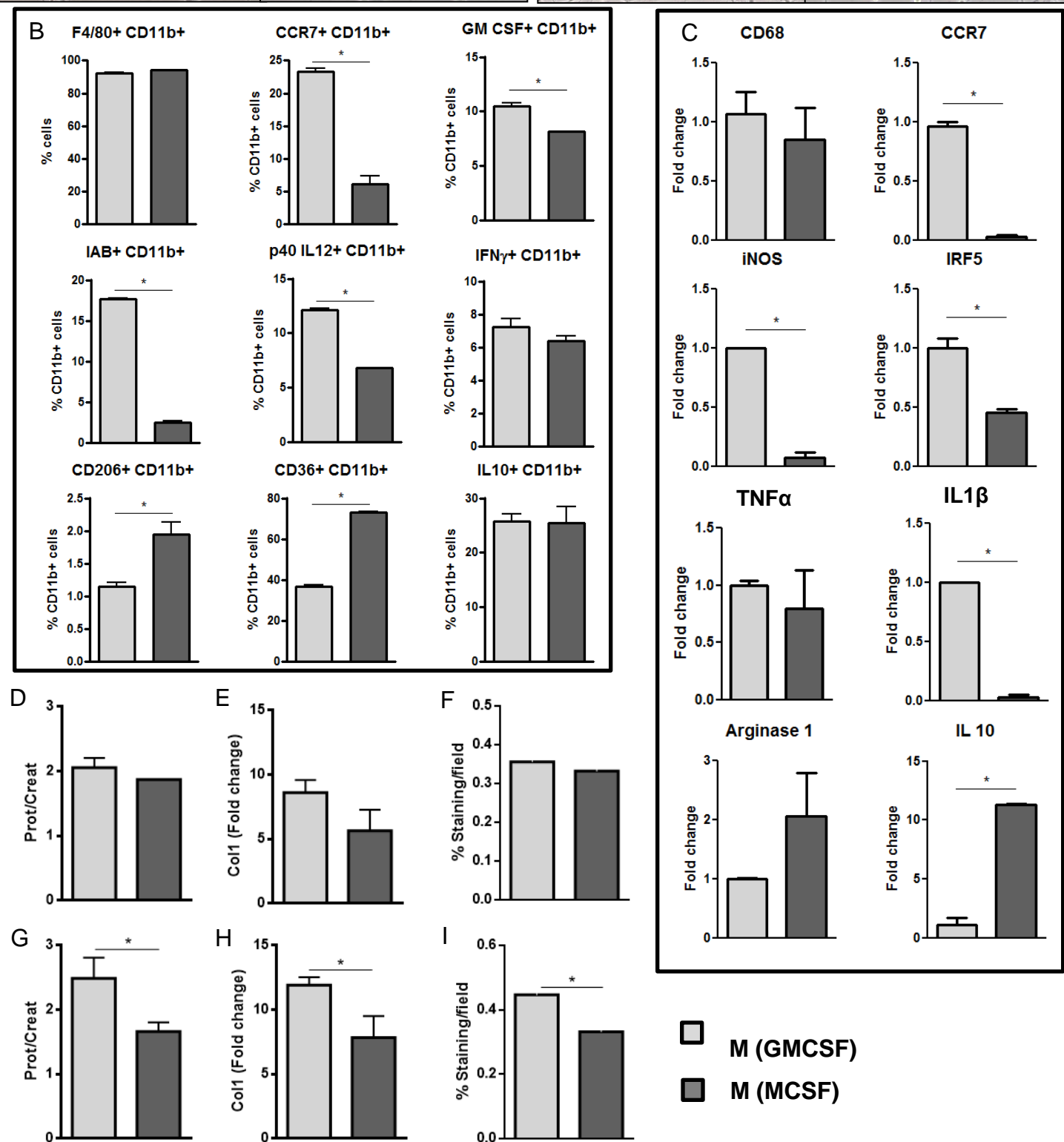

Sup. Fig. 2

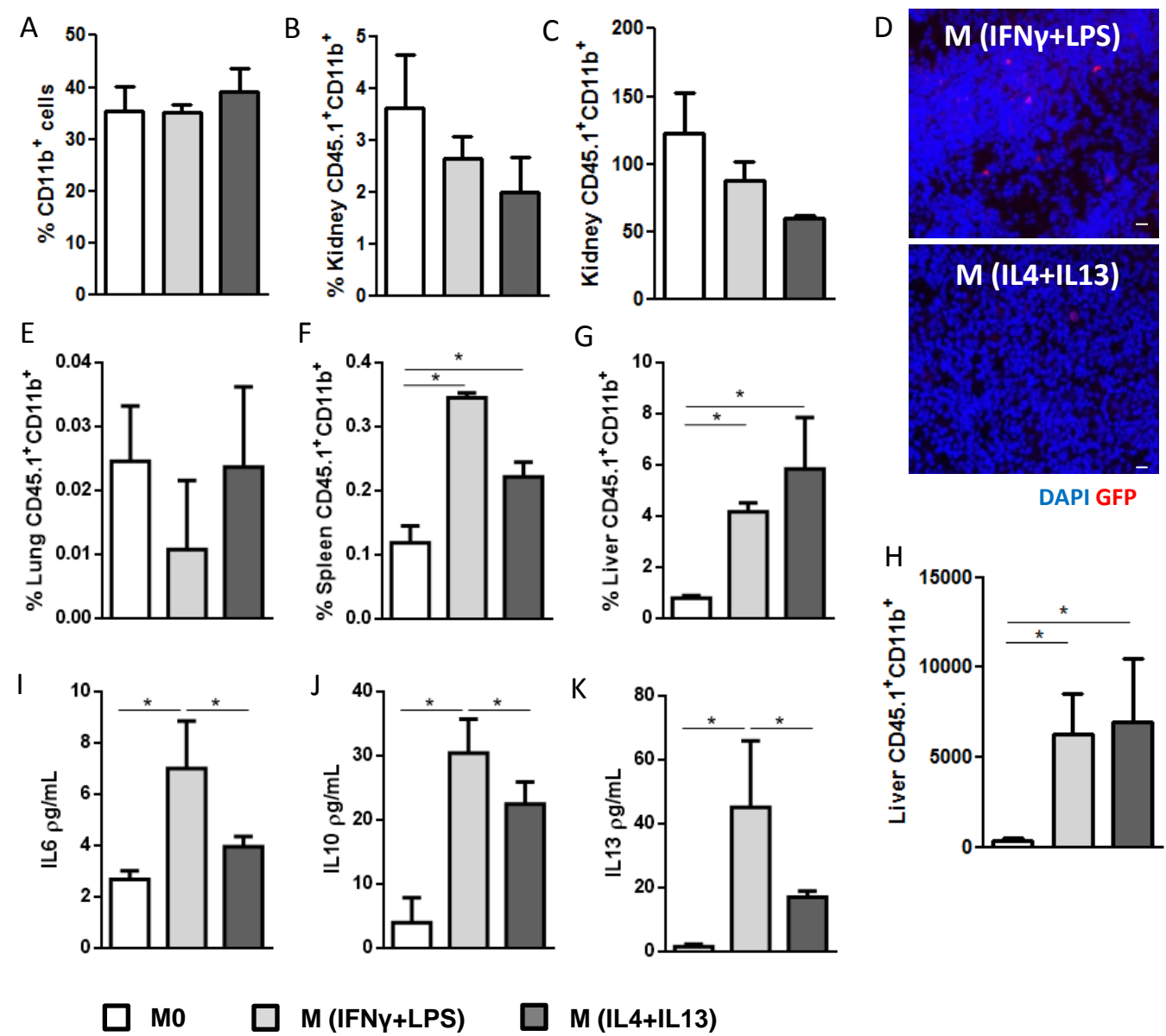

Sup. Fig. 3

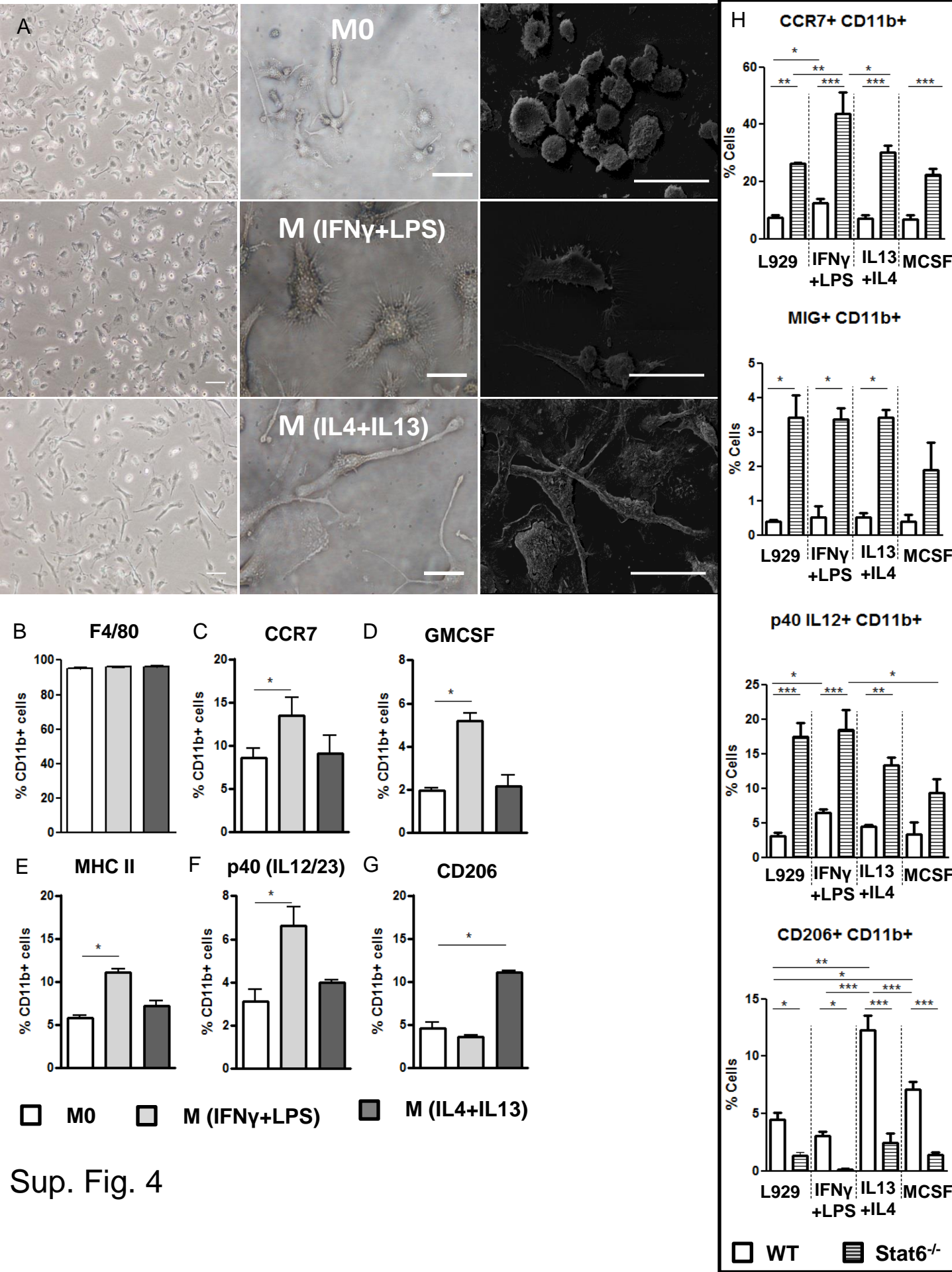

Sup. Fig. 4

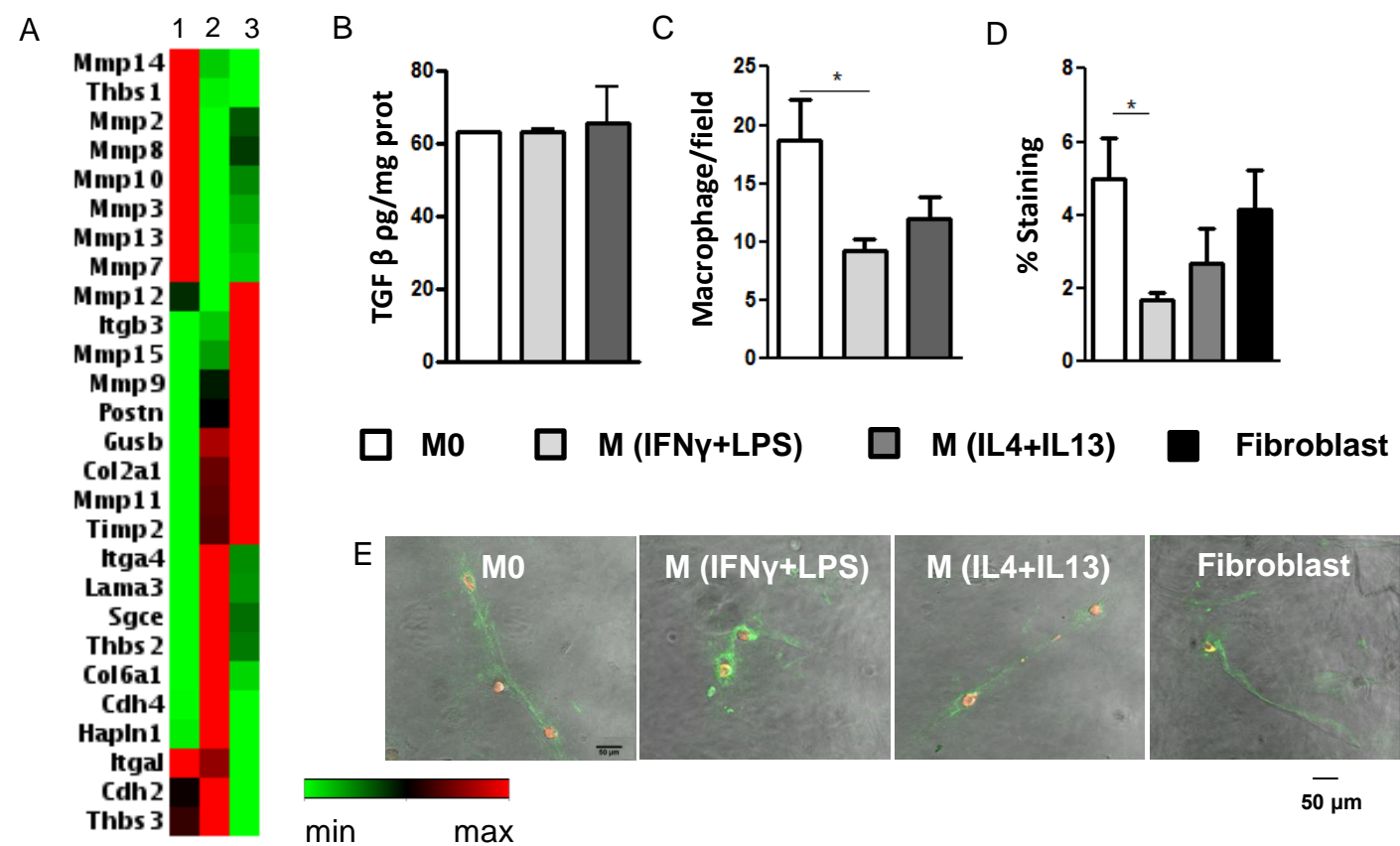

Sup. Fig. 5

Supplement: Supplementary file 1 — Figure S1. In vitro and ex vivo sorted CCR7+CD11b+ cell injection accentuates obstructive kidney fibrosis compared with CD206+CD11b+ cell injection. Figure S2. M (GMCSF) macrophage injection accentuates obstructive kidney fibrosis compared with M (MCSF) injection. Figure S3. M (IFNγ + LPS) macrophages induce inflammation and fibrosis in an endocrine manner. Figure S4. M0, M (IFNγ + LPS) and M (IL4 + IL13) characterization. Figure S5. M (IFNγ + LPS) and M (IL4 + IL13) macrophages present decreased migratory ability. [file IID3-4-300-s001.pdf]
